# Sleep health as a predictor of the course of depressed mood and loss of interest in individuals with depression

**DOI:** 10.1016/j.ijchp.2025.100653

**Published:** 2025-11-27

**Authors:** Sarah R. Schmid, Julian E. Schiel, Bernd Feige, Florian Holub, Elisabeth Hertenstein, Katharina Domschke, Martin K. Rutter, Kai Spiegelhalder

**Affiliations:** a Department of Psychiatry and Psychotherapy, Medical Centre – University of Freiburg, Faculty of Medicine, University of Freiburg, Freiburg, Germany; b German Center for Mental Health (DZPG), Partner Site Berlin/Potsdam, Berlin, Germany; c Department of Psychiatry, Faculty of Medicine, University of Geneva, Geneva, Switzerland; d Centre for Biological Timing, Faculty of Biology, Medicine and Health, University of Manchester, UK; e Diabetes, Endocrinology and Metabolism Centre, Manchester University NHS Foundation Trust, Manchester Academic Health Science Centre, Manchester, UK

**Keywords:** Sleep health, Depression, Insomnia, Daytime sleepiness, Chronotype, Longitudinal

## Abstract

**Background:**

Sleep health is a significant risk factor for incident depression. Much less is known about the impact of sleep health on the course of depressive symptoms in those who have already developed the disorder. The present study used longitudinal data of individuals with a depression diagnosis from the UK Biobank to analyse sleep health as a potential predictor for the course of depressive symptoms. The hypothesis was that better sleep health would predict a milder course of depressed mood and loss of interest.

**Methods:**

Self-reported insomnia symptoms, sleep duration, chronotype and daytime sleepiness were assessed as predictors at baseline. Depressive symptoms were self-reported and assessed at baseline and at follow-up, 8.76 ± 3.00 years after the baseline assessment. Linear regression models were calculated for each outcome variable.

**Results:**

The study comprised 4817 participants. Frequent insomnia symptoms and daytime sleepiness predicted a worse course of depressed mood (insomnia: β = 0.31 ± 0.10, *p* = 0.002; daytime sleepiness: β = 0.45 ± 0.11, *p* < 0.001) and loss of interest (insomnia: β = 0.30 ± 0.11, *p* = 0.005; daytime sleepiness: β = 0.32 ± 0.11, *p* = 0.003). On the contrary, early chronotype (β = -0.27 ± 0.10, *p* = 0.008) predicted a milder course of loss of interest.

**Conclusion:**

Our findings suggest that sleep health variables have a significant impact on the course of depressive symptoms in a sample with clinically diagnosed depression. Future research may investigate the add-on effects of sleep health improvements in patients with depression receiving guideline-concordant treatment.

## Introduction

Depression is the leading mental health concern causing disability-adjusted life years worldwide (Herrman et al., 2019). An estimated 5 % of the global adult population fulfil the diagnostic criteria for a depressive disorder with women being more frequently affected than men (6 % vs. 4 %; World Health Organization, 2023). Approximately one-third to one-half of major depressive disorders persist, or persist partially, despite treatment with multiple antidepressants (Marwaha et al., 2023). The high prevalence rates of depression are also reflected in significant health economic costs (Linder et al., 2020; Olesen et al., 2012). Thus, identifying conditions contributing to the development and maintenance of depression has been the subject of substantial research. In this regard, impairment in ‚sleep health‘ may be among the most important risk factors.

The emerging concept of ‚sleep health‘ was introduced by Daniel J. Buysse in 2014 and represents a positive, holistic view of sleep as a multidimensional phenomenon (Buysse, 2014). Sleep continuity, sleep duration, sleep timing, daytime sleepiness, and sleep satisfaction have been suggested to be the most relevant dimensions of sleep health as all of these are closely linked to physical and psychological well-being (Buysse, 2014; Hale et al., 2020).

Disturbances of sleep continuity (i.e., insomnia symptoms) have been demonstrated to be a risk factor for the onset of depression. A meta-analysis by Hertenstein and colleagues (2019) indicated that patients with insomnia have a substantially higher risk of developing depression compared to healthy, good sleepers (10 studies, OR: 2.83, *N* = 103,590). Furthermore, meta-analytical findings suggest that short sleep duration increases the risk of depression onset by around 40 % (10 studies, adjusted RR: 1.43, *N* = 19,968) compared to normal sleep duration (Zhang et al., 2024). Chronotype, as a proxy for sleep timing, is also related to depression onset (Lotti et al., 2022). More specifically, individuals with an evening preference are at higher risk (two studies, OR: 1.86; *N* = 4529). Regarding the underlying mechanism, findings indicate transdiagnostic genetic links between circadian endophenotypes, such as an evening preference, and phenotypes related to mood instability and neuroticism (Meyer et al., 2024). Besides variables directly related to night-time sleep, (excessive) daytime sleepiness is another dimension of sleep health that predicts the onset of depression (six studies, OR: 1.43, *N* = 22,162; Zhang et al., 2022).

These findings suggest that sleep health is a significant risk factor for incident depression. However, to our knowledge, no study has examined the relative impact of sleep health dimensions on the course of depressive symptoms (e.g., depressed mood and loss of interest) in adults already diagnosed with a depressive disorder. If the role of sleep health is equally important for the course of depression as for the new onset of the disorder, this would highlight the potential value of placing greater emphasis on sleep-related interventions (e.g., sleep restriction and stimulus control) in multimodal treatment programmes for depression.

In the current study, longitudinal data from individuals with a diagnosis of depression in the UK Biobank are used to analyse sleep health (i.e., insomnia symptoms, sleep duration, chronotype, daytime sleepiness) as a potential predictor of the course of depressive symptoms (i.e., depressed mood and loss of interest). We hypothesise that better sleep health will predict a milder course of depressive symptoms among patients diagnosed with a depressive disorder.

## Methods

### UK biobank population

The UK Biobank is a large epidemiological database that has been regularly updated with clinical outcome data since 2006. It contains data from approximately 500,000 participants from the UK, aged between 37 and 73 years at baseline (T0). The UK Biobank draws on clinical health records from the UK National Health Service as well as additional data collected directly at the study assessment centres. The aim of the UK Biobank is to provide data that will improve the prediction and management of diseases commonly experienced by middle-aged and elderly people (Sudlow et al., 2015). All participants were registered with the UK’s National Health Service (NHS) and gave written informed consent. The UK Biobank has ethical approval from the North West Multi-centre Research Ethics Committee (MREC; 21/NW/0157), which is renewed every five years.

For the present study, all UK Biobank participants reporting a neurological disease *(n = 22,084, see Supplementary Table S1)* or a sleep-related breathing disorder *(n = 1512)* at T0 were excluded to reduce confounding. In addition, all participants without a previous diagnosis of a depressive disorder at T0 (description below) and participants with incomplete data for any of the analysed variables were also excluded.

### ICD-10 depression diagnosis

Data of first occurrences of depressive episodes (ICD-10 F32.0 to F32.9; UK Biobank data field: 130,894) and recurrent depressive disorders (ICD-10 F33.0 to F33.9; UK Biobank data field: 130,896) were examined. Only patients who received one of these diagnoses prior to T0 were included in this study. Data on first occurrences of depressive episodes and depressive disorders were generated from multiple sources, including primary care data, hospital inpatient records, and participants’ self-reports of a prior ICD-10 depression diagnosis made by a medical doctor at T0. In cases where multiple data sources reported the same mental health condition, the earliest recorded date was used to determine the onset of the disorder (see https://biobank.ndph.ox.ac.uk/showcase/refer.cgi?id=593).

### Course of depressed mood and loss of interest

Depressive symptoms were operationalised by the self-reported current levels of depressed mood (UK Biobank data field: 2050) and loss of interest (UK Biobank data field: 2060) assessed at T0 (between 2006 and 2010) and T1 (between 2012 and 2013) or T2 (between 2014 and 2020). The primary endpoint of this study is depressive symptoms (i.e., depressed mood and loss of interest) at T1 or T2 adjusted for baseline levels. In individuals for whom follow-up data were available at both T1 and T2, data from the longer follow-up period (T2) were used. The variables were assessed by the following questions: "Over the past two weeks, how often have you felt down, depressed or hopeless?" and “Over the past two weeks, how often have you had little interest or pleasure in doing things?” with the response options: “not at all”, “several days”, “more than half the days”, and “nearly every day”. The response options were transformed into the number of days with depressive symptoms in the last two weeks (not at all = 0 days; several days = 4 days; more than half the days = 8 days; nearly every day = 13 days).

### Sleep health dimensions

Sleep health was assessed at T0 and operationalised using UK Biobank variables representing the most relevant components of this concept. In order to allow for comparability between studies, the same variables were used as in earlier UK Biobank studies on sleep health (Ell et al., 2023; Schiel et al., 2022).

*Sleep continuity* was evaluated by asking the participants the following question: "Do you have trouble falling asleep at night or do you wake up in the middle of the night?" with response options: “never/rarely”, “sometimes”, and “usually” (UK Biobank data field: 1200). Participants who responded "usually" were classified as having frequent insomnia symptoms, while those who selected one of the other two options were categorised as not having frequent insomnia symptoms.

Data on *sleep duration* were collected using the question: "About how many hours sleep do you get in every 24 h? (please include naps)" (UK Biobank data field: 1160). Due to the U-shaped relationship between sleep duration and many health outcomes (Alvarez & Ayas, 2004), the variable was categorised into short (< 7 h), normal (7–9 h), and long (> 9 h) sleep duration following current guidelines (Watson et al., 2015).

*Chronotype* (i.e., daytime preference) was used as a proxy for the sleep health dimension of sleep timing and assessed by asking the participants: "Do you consider yourself to be?", with the response options: “definitely a ‘morning’ person”, “more a ‘morning’ than ‘evening’ person”, “more an ‘evening’ than a ‘morning’ person”, and “definitively an ‘evening’ person” (UK Biobank data field: 1180). The two middle response options were subsumed under the category ‘intermediate chronotype’.

*Daytime sleepiness* was assessed by the question "How likely are you to doze off or fall asleep during the daytime when you don't mean to? (e.g., when working, reading or driving)" with the response options: “never/rarely”, “sometimes”, “often”, and “all of the time” (UK Biobank data field: 1220). Participants who selected the first option were categorised as having 'low daytime sleepiness', while those who chose one of the last three options were categorised as having 'high daytime sleepiness'.

### Medication

Baseline medication at T0 was self-reported to a research nurse. Participants were dichotomised according to whether or not they were taking sleep medication (i.e. benzodiazepines and benzodiazepine receptor agonists) or any other psychotropic medication (mood stabilisers, antidepressants, and antipsychotics; *see Supplementary Table S2* and *Supplementary Tables S3/1* and *Table S3/2*).

### Demographic data

Demographic data were assessed at T0. It comprised sex, age, educational level, ethnicity and socioeconomic status measured by the Townsend index of material deprivation (Townsend et al., 1988) (UK Biobank data fields: 31; 21,003; 6138; 21,000; 22,189). Socioeconomic status was log-transformed due to a skewed distribution using a ‘ln (*x* + 7)’ equation (minimum of non-transformed index: −6.18). Education levels were dichotomised into ‘holding a college/university degree’ or ‘not holding a college/university degree’. Ethnicity was categorised into six subgroups (*see Supplementary Table S4*).

### Psychosocial data

Psychosocial data were self-reported and assessed at T0. They comprised partnership status, loneliness, employment status, average total household income and level of worrying (UK Biobank data fields: 6141; 2020; 20,119; 748; 1980). Partnership status was categorised as ‘living with a partner’ or ‘not living with a partner’. Loneliness was categorised in ‘feeling lonely’ or ‘not feeling lonely’. Employment status was categorised in ‘being employed’ or ‘being unemployed’. Total household income was classified into five income level groups (*see Supplementary Table S5*). Level of worrying was categorised in ‘being a worrier’ or ‘not being a worrier’.

### Statistical analyses

For descriptive purposes, proportions were calculated for categorical variables and means, and standard deviations were calculated for continuous variables at T0. Questionnaire response options “do not know” and “prefer not to answer” were treated as missing values. Two linear regression models were used to predict depressed mood and loss of interest at follow-up. Multicollinearity was examined prior to analysis, with a VIF of maximum 2.3 for depressed mood and loss of interest and a maximum of 1.5 for all other predictors, indicating that multicollinearity was not a matter of concern. Baseline values of sleep health variables were entered as predictors in these models with ‘normal sleep duration’ and ‘intermediate chronotype’ as reference categories for sleep duration and chronotype. Sleep and psychotropic medication intake, demographic data (sex, age, educational level, ethnicity, socioeconomic status), psychosocial data (partnership status, loneliness, employment status, average total household income and level of worrying)*,* as well as depressed mood and loss of interest at T0 were used as covariates. Covariates were selected based on previously reported associations with depression severity (Buckman et al., 2018; Malhi & Mann, 2018; Schramm et al., 2020; Struijs et al., 2018).

Backward elimination based on Akaike’s Information Criterion (Akaike, 1974) was used for each model to reduce multicollinearity and to identify the model with the highest statistical power. Resulting coefficients of all models are reported as β ± standard error. After Bonferroni correction, the alpha level was set at *p* < 0.025 for the two primary analyses. All statistical analyses were conducted using the statistical software R, version 4.4.1.

## Results

### Sample description

The study comprised 4817 participants (64.0 % female, age 54.3 ± 7.3 years) with a diagnosis of depression. Medical and sociodemographic characteristics of the sample are presented in Table 1.

**Table 1:** Description of covariates at baseline.

Covariate	Description
Sleep medication use (n, %)	98 (2.0 %)
Psychiatric medication use (n, %)	1483 (30.8 %)
Ethnicity (n, %)	
*White*	4716 (97.9 %)
*Mixed*	35 (0.7 %)
*Asian or Asian British*	20 (0.4 %)
*Black or Black British*	14 (0.3 %)
*Chinese*	5 (0.1 %)
*Other*	27 (0.6 %)
College/university degree (n, %)	2173 (45.1 %)
Employed (n, %)	3813 (66.1 %)
Household income (mean, SD)	42,710 £ (29,385 £)
Socioecomic status* (mean, SD)	1.54 (0.54)
Living with a partner (n, %)	1574 (32.7 %)
Feeling lonely (n, %)	1593 (33.1 %)
Being a worrier (n, %)	3564 (74.0 %)

*Townsend deprivation index after log-transformation. SD: standard deviation.

### Description of sleep health variables

Regarding the sleep health variables, 1659 participants (34.4 %) reported frequent insomnia symptoms, 1172 participants (24.3 %) reported short sleep duration, 3513 participants (72.9 %) reported normal sleep duration and 132 participants (2.7 %) long sleep duration. Daytime sleepiness was reported by 1185 participants (24.6 %). Regarding chronotype, 1049 participants (21.8 %) reported being an early chronotype, 3136 participants (65.1 %) reported being an intermediate type, and 632 participants (13.1 %) reported being a late chronotype. At T0, the mean number of days with depressed mood was 2.8 days (SD: 3.8 days), at follow-up 2.3 days (SD: 3.5 days). The mean number of days with loss of interest at T0 was 2.3 days (SD: 3.5 days) and at follow-up 2.1 days (SD: 3.5 days). Mean time between baseline and follow-up assessments was 3197 days (SD: 1096 days).

### Course of depressed mood and loss of interest

**Fig. 1:**
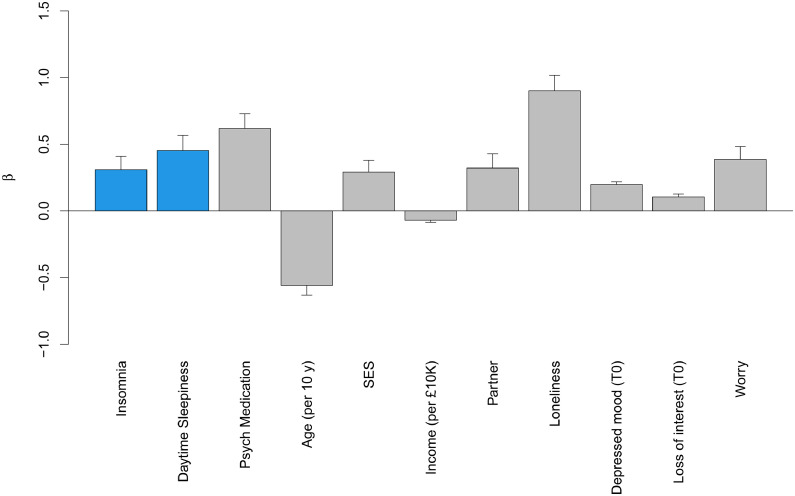
Relationship of significant sleep health variables/ covariates that were included in the final model after backward elimination with the number of days with depressed mood over the preceding two weeks as assessed at follow-up. The Y-axis shows the beta values from linear regression models. Bars representing relationships with sleep health variables are presented in blue, bars representing relationships with covariates in grey. Level of significance was set to p < 0.025. Error bars show the standard error of β. Psych medication = psychotropic medication; SES = socioeconomic status.

**Fig. 2:**
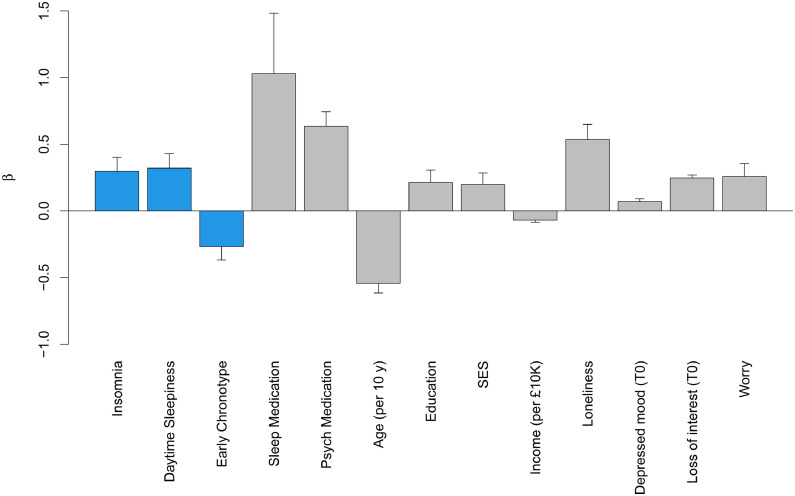
Relationship of significant sleep health variables/ covariates that were included in the final model after backward elimination with the number of days with loss of interest over the preceding two weeks as assessed at follow-up. The Y-axis shows the beta values from linear regression models. Bars representing relationships with sleep health variables are presented in blue, bars representing relationships with covariates in grey. Level of significance was set to p < 0.025. Error bars show the standard error of β. Psych medication = psychotropic medication; SES = socioeconomic status.

Full results of the final models after backward elimination are presented in Fig. 1, Fig. 2, and in the *Supplementary Table S6*.

### Depressed mood

Two sleep health dimensions were significant predictors of the number of days in the preceding two weeks that participants had felt down, depressed or hopeless, after adjusting for baseline symptom level and other covariates. Frequent insomnia symptoms compared to no frequent insomnia symptoms (β = 0.31 ± 0.10, *p* = 0.002) and daytime sleepiness compared to no daytime sleepiness (β = 0.45 ± 0.11, *p* < 0.001) predicted a worse course of depressed mood. After backward elimination, the final model did not include any of the other sleep health dimensions.

### Loss of interest

Three of the sleep health dimensions emerged as significant predictors of the number of days in the preceding two weeks that participants had little interest or pleasure in doing things, after adjusting for baseline symptom level and other covariates. Frequent insomnia symptoms compared to no frequent insomnia symptoms (β = 0.30 ± 0.11, *p* = 0.005) and daytime sleepiness compared to no daytime sleepiness (β = 0.32 ± 0.11, *p* = 0.003) predicted a worse course of loss of interest. In contrast, early chronotype compared to intermediate chronotype predicted a milder course of loss of interest (β = −0.27 ± 0.10, *p* = 0.008). Late chronotype compared to intermediate chronotype did not reach statistical significance (β = 0.30 ± 0.16, *p* = 0.052). Short sleep duration (β = 0.25 ± 0.12, *p* = 0.032) and long sleep duration (β = 0.35 ± 0.36, *p* = 0.333) compared to normal sleep duration did not reach the level of significance but were included in the final model after backward elimination.

## Discussion

The present study aimed to investigate the relationship between sleep health with the course of the core symptoms of depression (i.e., depressed mood and loss of interest) in patients diagnosed with a depressive disorder. The results indicate that, after controlling for confounding variables, frequent insomnia symptoms and daytime sleepiness are negatively related to the course of depressive symptoms, while an early chronotype is positively related. In contrast, subjectively reported habitual sleep duration did not predict the course of depressive symptoms, when controlling for other sleep health dimensions.

Frequent insomnia symptoms were negatively related to the course of both depressed mood and loss of interest in the current study. This suggests that the improvement of sleep continuity could be a reasonable aim in the overall management of depression. According to current guidelines, cognitive behavioural therapy for insomnia (CBT-I) is the first-line treatment for insomnia based on decades of research demonstrating its efficacy (Morin et al., 2024; Riemann et al., 2023; van Straten et al., 2018). Importantly, more recent meta-analyses have also shown that CBT-I improves symptoms of depression beyond the mere reduction of insomnia symptoms when compared to active and passive control conditions (Benz et al., 2020; Furukawa et al., 2024; Hertenstein et al., 2022). Thus, the overall benefits of including CBT-I techniques in the standard management of depression in routine clinical care should be more rigorously tested in future clinical trials.

In contrast to these considerations, the intake of sleep medication (i.e., benzodiazepines and benzodiazepine receptor agonists), was negatively related to the course of loss of interest in the present study. This result is in line with other reports on detrimental effects of benzodiazepines and benzodiazepine receptor agonists in patients with depression (Fond et al., 2023; Jeong et al., 2020) and guidelines which discourage long-term benzodiazepine therapy in these patients (Kassenärztliche Bundesvereinigung & Versorgungsleitlinie, 2022; NICE, 2022). Another potential explanation for the relationship between sleep medication use and the course of loss of interest is that individuals with severe depression are more likely to take sleep medication than those with mild symptoms. Therefore, reverse causality cannot be ruled out.

Daytime sleepiness was also a strong predictor of an adverse course of depressed mood and loss of interest in UK Biobank participants with depression. These results suggest that daytime sleepiness may also deserve more clinical attention in the treatment of depression. Since daytime sleepiness is a common symptom that is associated with a wide range of sleep, psychiatric and medical disorders, it usually requires an in-depth diagnostic exploration (Pérez-Carbonell et al., 2022). For example, while known cases of sleep apnoea were excluded in the current study, the astonishingly low prevalence of sleep apnoea in the UK Biobank suggests that there may be many undiagnosed cases in this sample that would benefit from appropriate treatment (see Senaratna et al., 2017 for comparison). Further research on daytime sleepiness is also needed to develop an efficient diagnostic workflow and improve available treatment options for specific underlying conditions.

Being an early chronotype was positively related to the course of loss of interest. One possible explanation is that the circadian system of early chronotypes is better aligned with environmental demands than that of intermediate and late chronotypes. In particular, these individuals may get enough sleep before working days, potentially leading to higher work productivity and job satisfaction (Roenneberg et al., 2012). Chronotherapeutic treatments (e.g., light therapy) or timed melatonin intake may be investigated when circadian alignment is not achieved (Morgenthaler et al., 2007).

Surprisingly, subjectively reported sleep duration did not emerge as a significant predictor of the course of depressive symptoms for either of the outcome parameters (i.e., depressed mood and loss of interest). This is at odds with the established role of sleep duration as a predictor of the new onset of depression (Firth et al., 2020; Zhang et al., 2024). Thus, it appears that sleep duration may be a reasonable target for the prevention of depression but less important for the management of depressed mood and loss of interest in an already diagnosed disorder.

Since sleep restriction therapy leads to continuous, uninterrupted sleep but has no or even temporarily negative effects on sleep duration (Kyle et al., 2014; Maurer et al., 2021), this may further support the use of sleep restriction therapy, one of the most effective components of CBT-I (Furukawa, Sakata et al., 2024; Steinmetz et al., 2024).

Some limitations of the current study need to be addressed. First, observational epidemiological studies do not allow conclusions regarding causality. Given that insomnia is a common symptom of depression and that depression can also predict later daytime sleepiness (Jaussent et al., 2017), reverse causality cannot be ruled out. Furthermore, the variables assessed in the present study (e.g., depressed mood and loss of interest) are based on single-item self-report questions, a general limitation in many epidemiological studies, which increases, e.g., susceptibility to measurement errors and decreases, e.g., sensitivity to detect subtle differences or to detect changes over time. Additionally, the sample is relatively healthy compared to the general population and is restricted to a specific age range (37-73 years) (Davis et al., 2020; Fry et al., 2017). This may limit the generalisability of the findings. The key strengths of our study are its large sample size and the resulting high statistical power.

In summary, sleep health variables are significantly related to the course of depressed mood and loss of interest in a clinically diagnosed sample of patients with depression. These findings underscore the importance of integrating strategies to improve sleep health into the clinical management of depression. Furthermore, the results also suggest that some sleep health variables, in particular subjective sleep duration, are less important for the course of an already diagnosed depressive disorder. Future clinical trials should investigate the added benefits of improving sleep health in patients with depression receiving guideline-concordant treatment. Additionally, increasing the frequency of assessments and the number of depressive symptoms measured would yield more precise data on the course of depressive symptoms and should be considered in future studies.

## Declaration of generative AI and AI-assisted technologies in the manuscript preparation process

During the preparation of this work the authors used Chat GPT (open AI) in order to check spelling. After using this tool, the authors reviewed and edited the content as needed and take full responsibility for the content of the published article.

## Funding

This research did not receive any specific grant from funding agencies in the public, commercial, or not-for-profit sector.

## Declaration of competing interest

The authors declare that they have no known competing financial interests or personal relationships that could have appeared to influence the work reported in this paper.

## Appendix Supplementary materials

Image, application 1
