# Supplementary material for: Sleep health as a predictor of the course of depressed mood and loss of interest in individuals with depression
Source: Int J Clin Health Psychol. 2025 Nov 27;25(4):100653. doi: 10.1016/j.ijchp.2025.100653 (PMC12702323; doi:10.1016/j.ijchp.2025.100653)
Supplement: Supplementary file 1 [file mmc1.docx]

*Supplementary Materials*

**Sleep health as a predictor of the course of depressed mood and loss of interest in individuals with depression**

Sarah R. Schmid^1^, Julian E. Schiel^1^, Bernd Feige^1^, Florian Holub^1^, Elisabeth Hertenstein^3^, Katharina Domschke^1,2^, Martin K. Rutter^4,5^,
Kai Spiegelhalder^1^

1. Department of Psychiatry and Psychotherapy, Medical Centre – University of Freiburg, Faculty of Medicine, University of Freiburg, Freiburg, Germany
2. German Center for Mental Health (DZPG), Partner Site Berlin/Potsdam, Berlin, Germany
3. Department of Psychiatry, Faculty of Medicine, University of Geneva, Geneva, Switzerland
4. Centre for Biological Timing, Faculty of Biology, Medicine and Health, University of Manchester, UK
5. Diabetes, Endocrinology and Metabolism Centre, Manchester University NHS Foundation Trust, Manchester Academic Health Science Centre, Manchester, UK

**Address for correspondence:**

Sarah R. Schmid, M.Sc.

Department of Psychiatry and Psychotherapy

Medical Center – University of Freiburg

Hauptstraße 5, 79104 Freiburg, Germany

Tel: +49 761 270 65970

Fax: +49 761 270 66190

Email: sarah.schmid@uniklinik-freiburg.de

List of Tables

Table S1: List of neurological diseases

Table S2: Sleep medication – List of Hypnotics and Sedatives

Table S3: Psychotropic medication – List of Mood Stabilisers, Antidepressant and
 Antipsychotics

Table S4: Categorisation of ethnicity

Table S5: Categorisation of household Income

Table S6: Strength of relationships between sleep health variables and depressed mood or
 loss of interest, respectively after accounting for covariates in the final backward
 elimination models.

Table S1: *List of neurological diseases*. Data field is a fundamental block of data stored in the UK Biobank repository and refers to the results of a single item, measurement or outcome (or part thereof). Code is used for categorial variables and represents the actual data within a data field. For example, the Data Field 20001 refers to self-reported cancer illness and the Code 1031 represents Meningeal cancer/ Malignant meningioma. For more information see <https://biobank.ndph.ox.ac.uk/showcase/search.cgi>

| **Data Field** | **Code** | **Description** |
| --- | --- | --- |
| 6150 | 3 | Stroke |
| 20001 | 1031  1032 | Meningeal cancer / Malignant meningioma  Brain cancer / Primary malignant brain tumour |
| 20002 | 1491  1245  1425  1433  1258  1263  1246  1264  1266  1244  1583  1659  1247  1259  1261  1240  1683  1397  1434  1262  1524  1086  1083  1082 | Brain haemorrhage  Brain abscess / Intracranial abscess  Cerebral aneurysm  Cerebral palsy  Chronic / Degenerative neurological problem  Dementia / Alzheimer’s disease / Cognitive impairment  Encephalitis  Epilepsy  Head injury  Infection of nervous system  Ischaemic stroke  Meningioma/ Benign meningeal tumour  Meningitis  Motor neurone disease  Multiple Sclerosis  Neurological injury / trauma  Benign Neuroma  Other demyelinating disease (not multiple sclerosis)  Other neurological problem  Parkinson’s disease  Spina bifida  Subarachnoid haemorrhage  Subdural haemorrhage / Haematoma  Transient ischaemic attack (TIA) |

Table S2: *Sleep medication – List of Hypnotics and Sedatives*. Data field is a fundamental block of data stored in the UK Biobank repository and refers to the results of a single item, measurement or outcome (or part thereof). Code is used for categorial variables and represents the actual data within a data field. For example, the Data Field 20003 refers to Treatment/Medication code and the Code 1140863152 represents Diazepam. For more information see <https://biobank.ndph.ox.ac.uk/showcase/search.cgi>

| **Data Field** | **Code** | **Description (substance / trade name)** |
| --- | --- | --- |
| 20003 | 1140863152  1141157496  1140863244  1140863250  1140855856  1140863202  1140863210  1140863138  1140863144  1140928004  1141171404  1141171410  1140865016  1140864916  1140863182  1140863194  1140855896  1140863196  1140855900  1140855898  1140855902  1140855904  1140863104  1140863106  1140855914  1140855920 | Diazepam  Diazepam product  Valium 2 mg tablet  Valium 2 mg / 5 ml syrup  Valium 10 mg suppository  Temazepam  Normison 10 mg capsule  Euhypnos 10 mg / 5 ml oral solution  Zopiclone  Zimovane ls 3.75 mg tablet  Zaleplon  Sonata 5 mg capsule  Zolpidem  Stilnoct 5 mg tablet  Nitrazepam  Mogadon 5 mg tablet  Nitrados 5 mg tablet  Remnos 5 mg tablet  Somnite 5 mg tablet  Noctesed 5 mg tablet  Surem 5 mg capsule  Unisomnia 5 mg tablet  Flunitrazepam  Rohypnol 1 mg tablet  Triazolam  Halcion 125 micrograms tablet |

Table S3/1: *Psychotropic medication – List of Mood Stabilisers, Antidepressants, and Antipsychotics.* Data field is a fundamental block of data stored in the UK Biobank repository and refers to the results of a single item, measurement or outcome (or part thereof). Code is used for categorial variables and represents the actual data within a data field. For example, the Data Field 20003 refers to Treatment/Medication code and the Code 1140867490 represents Lithium. For more information see <https://biobank.ndph.ox.ac.uk/showcase/search.cgi>

| **Data Field** | **Code** | **Description (substance / trade name)** |
| --- | --- | --- |
| 20003 | 1140867490  1140867504  1140867494  1140872198  1140872200  1141172838  1140872214  1140872064  2038459704  1140872072  1141167860  1141185460  1141162898  1140864452 | Lithium product  Priadel 200 mg m/r tablet  Camcolit 250 tablet  Sodium valproate  Epilim 100 mg crushable tablet  Depakote 250 mg e/c tablet  Valproic acid  Carbamazepine product  Carbamazepine  Tegretol 100 mg tablet  Teril cr 200 mg m/r tablet  Teril retard 200 mg m/r tablet  Timonil retard 200 mg m/r tablet  Epimaz 100 mg tablet |
|  | 1140867888  1140882236  1140879540  1140867876  1140921600  1141151946  1141180212  1141190158  1140867878  1140867884  1140879544  1141152732  1141152736  1141200564  1141201834  1141200570  1140916282  1140916288  1140879616  1140867658  1140867668  1140867662  1140867948  1140867934  1140867938  1140856186  1140867928  1140867850  1140910704  1140867852  1140867920  1140867922  1140879630  1140867712  1140867756  1140867758  1140879628 | Paroxetine  Seroxat 20 mg tablet  Fluoxetine  Prozac 20 mg capsule  Citalopram  Cipramil 10 mg tablet  Escitalopram  Cipralex 5 mg tablet  Sertraline  Lustral 50 mg tablet  Fluvoxamine  Mirtazapine  Zispin 30 mg tablet  Duloxetine  Cymbalta 30 mg gastro-resistant capsule  Yentreve 20 mg gastro-resistant capsule  Venlafaxine  Efexor 37.5 mg tablet  Amitriptyline  Elavil 10 mg tablet  Tryptizol 10 mg tablet  Lentizol 25 mg m/r capsule  Amitriptyline hydrochloride 10 mg + Perphenazine 2 mg tablet  Triptafen tablet  Amitriptyline 12.5 mg + Chlordiazepoxide 5 mg capsule  Limbitrol 10 mg capsule  Limbitrol 5 mg capsule  Phenelzine  MAOI / Phenelzine  Nardil 15 mg tablet  Moclobemide  Manerix 150 mg tablet  Imipramine  Tofranil 10 mg tablet  Trimipramine  Surmontil 10 mg tablet  Dothiepin |

Table S3/2: *Psychotropic medication – List of Mood Stabilisers, Antidepressants, and Antipsychotics.* Data field is a fundamental block of data stored in the UK Biobank repository and refers to the results of a single item, measurement or outcome (or part thereof). Code is used for categorial variables and represents the actual data within a data field. For example, the Data Field 20003 refers to Treatment/Medication code and the Code 1140909806 represents Dosulepin. For more information see <https://biobank.ndph.ox.ac.uk/showcase/search.cgi>

| **Data Field** | **Code** | **Description (substance / trade name)** |
| --- | --- | --- |
| 20003 | 1140909806  1140867624  1141171824  1140879620 | Dosulepin  Prothiaden 25 mg capsule  Thaden 25 mg capsule  Clomipramine |
|  | 1140867690  1140867726  1140882310  1141146062  1140879556  1140867806  1140867812 | Anafranil 10 mg capsule  Lofepramine  Gamanil 70 mg tablet  Lomont 70 mg / 5 ml s/f suspension  Mianserin  Bolvidon 10 mg tablet  Norval 10 mg tablet |
|  | 1140879658  1140910358  1140863416  1140867168  1140867184  1140867092  1140867398  1140882098  1140867456  1140867156  1140856004  1140909800  1140867150  1140867152  1140867952  1140882100  1140867342  1140867406  1140867414  1140867084  1140867086  1140868120  1140867244  1140879750  1140867312  1141152848  1141152860  1140867444  1141177762  1140928916  1141167976  1141195974  1141202024  1141153490  1141184742  1140867420  1140882320 | Chlorpromazine  CPZ / Chlorpromazine  Largactil 10 mg tablet  Haloperidol  Haldol 5 mg tablet  Serenace 500 micrograms capsule  Fluphenazine decanoate  Fluphenazine  Modecate 12.5 mg/0.5 ml oily injection  Moditen 1 mg tablet  Moditen enanthate 25 mg/ml injection  Flupentixol  Flupenthixol  Depixol 3 mg tablet  Fluanxol 500 micrograms tablet  Zuclopenthixol  Clopixol 2 mg tablet  Loxapine  Loxapac 10 mg capsule  Droperidol  Droleptan 10 mg tablet  Trifluoperazine  Stelazine 1 mg tablet  Thioridazine  Melleril 10 mg tablet  Quetiapine  Seroquel 25 mg tablet  Risperidone  Risperdal 0.5 mg tablet  Olanzapine  Zyprexa 2.5 mg tablet  Aripiprazole  Abilify 5 mg tablet  Amisulpride  Solian 100 mg/ml s/f oral solution  Clozapine  Clozaril 25 mg tablet |

Table S4: *Categorisation of ethnicity.* Data field is a fundamental block of data stored in the UK Biobank repository and refers to the results of a single item, measurement or outcome (or part thereof). For example, the data field 21000 refers to ethnic background. Each data field belongs to at least one origin category (e.g., population characteristics) and can be further divided into subcategories (e.g., sociodemographics). Description provides further information about the categories. For more information see <https://biobank.ndph.ox.ac.uk/showcase/search.cgi>

| **Data Field** | **Category** | **Description** |
| --- | --- | --- |
| 21000 | White | British, Irish or any other white background |
|  | Mixed | White and Black Caribbean, White and Black African, White and Asian, any other mixed background |
|  | Asian or Asian British | Indian, Pakistani, Bangladeshi, any other Asian background |
|  | Black or Black British | Caribbean, African, any other black background |
|  | Chinese |  |
|  | Any other ethnic group |  |

Table S5: *Categorisation of household income.* Data field is a fundamental block of data stored in the UK Biobank repository and refers to the results of a single item, measurement or outcome (or part thereof). For example, the data field 738 refers to household income. Each data field belongs to at least one origin category (e.g., population characteristics) and can be further divided into subcategories (e.g., sociodemographics). Description provides further information about the categories. For more information see <https://biobank.ndph.ox.ac.uk/showcase/search.cgi>

| **Data Field** | **Category (in** £**)** |
| --- | --- |
| 738 | < 9000 |
|  | 24500 |
|  | 41500 |
|  | 76000 |
|  | > 100000 |

Table S6: *Strength of relationships between sleep health variables and depressed mood or loss of interest, respectively after accounting for covariates in the final backward elimination models*. Significant effects (p < 0.025) of the predictors are marked bold. A positive ß value means that the specific variable predicts an increased number of days with depressed mood or loss of interest, while a negative ß value indicates that the specific variable predicts a decreased number of days with depressed mood or loss of interest. For example, for insomnia symptoms, a beta of 2.98 x 10^-1^ associated with depressed mood indicates that when compared to participants without insomnia symptoms at baseline, patients with insomnia symptoms experienced 0.298 units more days of depressed mood at follow-up after accounting for baseline score and other covariates included in the model. t = t-value, CI = confidence interval, AIC = Akaike Information Criterion. Dropping further variables will not produce a model with higher statistical explanatory power (with lower AIC).

|  | **Variables included in backward elimination model** | **ß** | | | | **SE** | | | **t** | **95% CI** | | | | **p** | | |
| --- | --- | --- | --- | --- | --- | --- | --- | --- | --- | --- | --- | --- | --- | --- | --- | --- |
| **Depressed mood** | **Insomnia symptoms** | | **3.10 x 10^-1^** | | **1.01 x 10^-1^** | | | **3.07** | | | **[1.12 x 10^-1^, 5.07 x 10^-1^]** | | | | | **0.002** |
| (AIC = 10965.61) | **Daytime Sleepiness** | | **4.54 x 10^-1^** | | **1.13 x 10^-1^** | | | **4.03** | | | **[2.33 x 10^-1^, 6.74 x 10^-1^]** | | | | | **<0.001** |
|  | Sleep medication | | 1.05 x 10^0^ | | 4.73 x 10^-1^ | | | 2.22 | | | [1.24 x 10^-1^, 1.98 x 10^0^] | | | | | 0.026 |
|  | **Psychiatric medication** | | **6.18 x 10^-1^** | | **1.11 x 10^-1^** | | | **5.55** | | | **[4 x 10^-1^, 8.37 x 10^-1^]** | | | | **<0.001** | |
|  | **Age** | | **-5.59 x 10^-2^** | | **7.20 x 10^-3^** | | | **-7.77** | | | **[-7 x 10^-2^, -4.18 x 10^-2^]** | | | | | **<0.001** |
|  | **Socioeconomic status** | | **2.91 x 10^-1^** | | **8.79 x 10^-2^** | | | **3.31** | | | **[1.19 x 10^-1^, 4.63 x 10^-1^]** | | | | **0.001** | |
|  | Employment status | | 1.93 x 10^-1^ | | 1.12 x 10^-1^ | | | 1.72 | | | [-2.69 x 10**^-2^**, 4.13 x 10**^-1^**] | | | | 0.085 | |
|  | **Household income** | | **-6.91 x 10^-6^** | | **1.69 x 10^-6^** | | | **-4.10** | | | **[-1.02 x 10^-5^, -3.61 x 10^-6^]** | | | | **<0.001** | |
|  | **Partnership** | | **3.22 x 10^-1^** | | **1.07 x 10^-1^** | | | **3.02** | | | **[1.13 x 10^-1^, 5.31 x 10^-1^]** | | | | **0.003** | |
|  | **Loneliness** | | **9.01 x 10^-1^** | | **1.17 x 10^-1^** | | | **7.69** | | | **[6.72 x 10^-1^, 1.13 x 10^0^]** | | | | **<0.001** | |
|  | **Depressed mood T0** | | **1.98 x 10^-1^** | | **2.13 x 10^-2^** | | | **9.27** | | | **[1.56 x 10^-1^, 2.4 x 10^-1^]** | | | | **<0.001** | |
|  | **Loss of interest T0** | | **1.05 x 10^-1^** | | **2.3 x 10^-2^** | | | **4.54** | | | **[5.94 x 10^-2^, 1.5 x 10^-1^]** | | | | **<0.001** | |
|  | **Worrying** | | **3.86 x 10^-1^** | | **9.72 x 10^-2^** | | | **3.97** | | | **[1.96 x 10^-1^, 5.77 x 10^-1^]** | | | | **<0.001** | |
| **Loss of interest** | **Insomnia symptoms** | | **2.98 x 10^-1^** | | **1.05 x 10^-1^** | | | 2.83 | | | **[[9.14 x 10^-2^, 5.04 x 10^-1^]** | | | | **0.005** | |
| (AIC = 10848.58) | Short sleep duration | | 2.47 x 10^-1^ | | 1.15 x 10^-1^ | | | 2.15 | | | [2.18 x 10**^-2^**, 4.72 x 10**^-1^**] | | | | 0.032 | |
|  | Long sleep duration | | 3.48 x 10^-1^ | | 3.59 x 10^-1^ | | | **0.97** | | | [-3.56 x 10**^-1^**, 1.05 x 10**^0^**] | | | | 0.333 | |
|  | **Daytime Sleepiness** | | **3.21 x 10^-1^** | | **1.09 x 10^-1^** | | | **2.95** | | | **[1.08 x 10^-1^, 5.35 x 10^-1^]** | | | | **0.003** | |
|  | **Early chronotype** | | **-2.67 x 10^-1^** | | **1.01 x 10^-1^** | | | **-2.64** | | | **[-4.65 x 10^-1^, -6.92 x 10^-2^]** | | | | **0.008** | |
|  | Late chronotype | | 3.00 x 10^-1^ | | 1.55 x 10^-1^ | | | **1.94** | | | **[-2.72 x 10^-3^, 6.04 x 10^-1^]** | | | | 0.052 | |
|  | **Sleep medication** | | **1.03 x 10^0^** | | **4.55 x 10^-1^** | | | **2.26** | | | **[1.37 x 10^-1^, 1.92 x 10^0^]** | | | | **0.024** | |
|  | **Psychiatric medication** | | **6.36 x 10^-1^** | | **1.09 x 10^-1^** | | | **5.84** | | | **[4.22 x 10^-1^, 8.49 x 10^^-1^]** | | | | **<0.001** | |
|  | **Age** | | **-5.43 x 10^-2^** | | **7.27 x 10^-3^** | | | **-7.47** | | | **[-6.85 x 10^-2^, -4 x 10^-2^]** | | | | **<0.001** | |
|  | **Education** | | **2.14 x 10^-1^** | | **9.26 x 10^-2^** | | | **2.31** | | | **[3.27 x 10^-2^, 3.96 x 10^-1^]** | | | | **0.021** | |
|  | **Socioeconomic status** | | **1.97 x 10^-1^** | | **8.66 x 10^-2^** | | | **2.28** | | | **[2.81 x 10^-2^, 3.67 x 10^-1^]** | | | | **0.022** | |
|  | Employment status | | 2.52 x 10^-1^ | | 1.14 x 10^-1^ | | | 2.22 | | | [2.95 x 10^-2^, 4.75 x 10^-1^] | | | | 0.026 | |
|  | **Household income** | | **-6.82 x 10^-6^** | | **1.75 x 10^-6^** | | | **-3.90** | | | **[-1.02 x 10^-5^, -3.39 x 10^-6^]** | | | | **<0.001** | |
|  | Partnership | | 2.06 x 10^-1^ | | 1.06x 10^-1^ | | | 1.94 | | | [-2.2 x 10**^-3^**, 4.15 x 10**^-1^**] | | | | 0.053 | |
|  | **Loneliness** | | **5.36 x 10^-1^** | | **1.13 x 10^-1^** | | | **4.75** | | | **[3.15 x 10^-1^, 7.58 x 10^-1^]** | | | | **<0.001** | |
|  | **Depressed mood T0** | | **7.01 x 10^-2^** | | **2.05 x 10^-2^** | | | **3.43** | | | **[3.00 x 10^-2^, 1.10 x 10^-1^]** | | | | **<0.001** | |
|  | **Loss of interest T0** | | **2.47 x 10^-1^** | | **2.33 x 10^-2^** | | | **10.59** | | | **[2.01 x 10^-1^, 2.93 x 10^-1^]** | | | | **<0.001** | |
|  | **Worrying** | | **2.59 x 10^-1^** | | **9.69 x 10^-1^** | | | **2.67** | | | **[6.89 x 10^-2^, 4.49 x 10^-1^]** | | | | **0.008** | |
|  | Time between T0 and T1 | | 5.87 x 10^-5^ | | 4.22 x 10^-5^ | | | 1.39 | | | [-2.41 x 10**^-5^**, 1.41 x 10**^-4^**] | | | | 0.165 | |
|  |  | | |  | | |  | | | | |  |  | |  | |
